# Anti-Müllerian hormone as a diagnostic tool to identify queens with ovarian remnant syndrome

**DOI:** 10.1177/1098612X221099195

**Published:** 2022-05-30

**Authors:** Ulrike Flock, Stine Fischer, Jasmin Weeger, Sven Reese, Beate Walter

**Affiliations:** 1 Clinic of Small Animal Surgery and Reproduction at the Centre for Clinical Veterinary Medicine, Faculty of Veterinary Medicine, LMU Munich, Munich, Germany; 2 Chair of Anatomy, Histology and Embryology, Department of Veterinary Sciences, Faculty of Veterinary Medicine, LMU Munich, Munich, Germany

**Keywords:** spaying, castration, oestrus, heat signs

## Abstract

**Objectives:**

Ovarian remnant syndrome (ORS) is suspected when heat signs occur in spayed individuals, but further diagnostic procedures are necessary to exclude other possible oestrogen sources, such as the adrenal gland or exogenous supplementation. Anti-Müllerian hormone (AMH), secreted by granulosa cells or Sertoli cells, serves to differentiate sexually intact from gonadectomised animals and has been described in dogs as a tool for diagnosing ORS. The aim of this study was to evaluate if AMH determination can be used to diagnose ORS in cats.

**Methods:**

AMH was measured with a chemiluminescence immunoassay in serum samples of 15 sexually intact, 9 spayed and 16 cats with a history of heat signs after spaying. Abdominal ultrasound (n = 13), vaginal smears (n = 7), progesterone measurement (n = 5) and laparotomy (n = 14) were used to determine the presence of ovarian tissue. After surgery, a histological examination of the obtained tissue was performed in the cats with suspected ORS.

**Results:**

In 15 cats with ORS the AMH serum concentrations were significantly higher than in spayed cats (n = 10; *P* = 0.025) and significantly lower than in sexually intact cats (n = 15; *P* = 0.001). Among the cats with ORS, the highest AMH serum concentrations were measured in the queens with cystic ovarian alterations and in one cat from which a whole ovary was obtained. The cat with the lowest AMH serum concentration had a simultaneous high progesterone serum concentration. Cats with ORS did not show any heat signs after surgical removal of the ovarian tissue.

**Conclusions and relevance:**

A single determination of AMH in blood serum is a useful diagnostic tool for the diagnosis of ORS in cats, regardless of the hormonal activity of the remnant ovarian tissue.

## Introduction

Ovarian remnant syndrome (ORS) in cats is a consequence of the incomplete removal of the ovaries during elective spaying.^1–5^ Although frequently discussed, there is little evidence that congenital ectopic ovarian tissue occurs in any domestic animal species.^6^ Affected animals usually develop heat signs up to several months or years after spaying. Complications such as uterine stump pyometra, ovarian tumours or even hyperandrogenism seem to be rare in cats and have only been documented in isolated case reports.^2,7–10^

Several diagnostic methods have been described to verify the presence of ovarian tissue. In addition to heat signs, an exfoliative vaginal cytology containing a high number of superficial cells or an increased serum oestradiol concentration may indicate oestrogen-secreting ovarian tissue. However, administration of exogenous oestrogen has to be excluded.^1^ After spontaneous or induced ovulation an elevated serum progesterone concentration confirms luteal tissue.^11,12^ When no heat signs are present during the time of examination, an oestrogen stimulation test or luteinising hormone (LH) test has been described.^13,14^ However, most of these diagnostic approaches have been solely tested in sexually intact queens; therefore, it can only be assumed that cats with ORS show comparable hormonal changes. Abdominal ultrasonography can detect remnant ovarian tissue, especially when follicles or corpora lutea are present.^5^

The preferred treatment is the surgical removal of the remnant ovarian tissue via laparotomy, although laparoscopic approaches have been described.^4,5,15^ In most cases, the remnant ovarian tissue is located at one or both of the ovarian pedicles, but ovarian tissue displaced to other locations inside the abdominal cavity during spaying has the potential to revascularise and resume its hormonal activity.^9,16^

Serum anti-Müllerian hormone (AMH), secreted from Sertoli cells in males and granulosa cells in females, helps to distinguish sexually intact dogs and cats from gonadectomised individuals.^17–19^ Furthermore, the usefulness of serum AMH concentration to diagnose ORS in female dogs has been described.^20,21^ AMH determination as a diagnostic tool to identify cats with remnant ovarian tissue has only been used in a few cases so far.^16,18^

The aim of this study was to determine the use of serum AMH to identify ORS in cats alone or in combination with other diagnostic approaches at different stages of hormonal activity of the remnant ovarian tissue.

## Material and methods

### Animals

This study included 15 cats, shown in Table 1, that were examined from January 2017 to October 2021, and were presented because of recurring oestrous behaviour after spaying, which took place between 1 and 69 months before presentation. In total, 13 of these cats were presented to our clinic and two to a private practitioner.All cats underwent a general clinical examination. An abdominal ultrasound was performed in 13 cats to examine if remnant ovarian tissue was present behind the kidneys at the position of the ovaries (excluding cats 4 and 13). A vaginal swab was obtained and stained (DiffQuik, RAL Diagnostics) in seven cats (cats 2, 3, 8, 9, 11, 14 and 15) and evaluated. We determined the amount and type of epithelial cells, the quality of the background, the presence of other cells such as neutrophils or erythrocytes and the presence of bacteria. In three cats (cats 2, 5 and 14) serum progesterone levels were determined at first clinical presentation and in a further two cats (cats 3 and 8) serum progesterone levels were determined 6 days after injection of 0.5 ml of human chorionic gonadotropin (hCG) (Ovogest 300 IE/ml; MSD Tiergesundheitsdienst) intramuscularly. With the exception of cat 7, all cats underwent a laparotomy under general anaesthesia and a histopathological examination of the removed tissue was performed afterwards. For general anaesthesia at our clinic, the cats received premedication including diazepam and ketamin for sedation and methadone for analgesia, and were induced with either propofol or alfaxalone. After intubation, anaesthesia was maintained with isoflurane. For laparotomy, a midline incision from behind the umbilicus to the height of the last pair of mammary glands was performed, and the areas behind both kidneys, as well as the remnant uterus, were investigated, and all ovarian-like structures were removed for pathohistological examination. For postoperative analgesia, the cats were treated with meloxicam for 3 days.

**Table 1:** Breed, age, body weight, time span between initial spaying and reoccurrence of heat signs, histology of the ovarian tissue and the anti-Müllerian hormone concentration of the cats with suspected ovarian remnant syndrome included in the study

Cat number	Breed	Age (months)	Body weight (kg)	Reoccurrence of oestrous signs after spaying (months)	Histology of the ovarian tissue	AMH (ng/ml)
1	BSH	28	7.5	2	Polycystic	1.63
2	BSH	12	3.2	2	Whole ovary; corpora lutea in regression and small follicles	1.52
3	BSH	39	3.5	9	Luteal cyst	1.13
4	ESH	41	2.9	Unknown	Simple cyst	0.92
5	Maine Coon	94	4.0	69	Polycystic	0.77
6	BSH	25	4.7	18	Polycystic	0.73
7	ESH	7	2.2	1	No histology	0.69
8	ESH	21	2.6	6	Corpora lutea and small follicles	0.6
9	ESH	60	4.2	12	Corpora lutea in regression and small follicles	0.51
10	Persian	36	3.2	9	Corpora lutea in regression and small follicles	0.43
11	ESH	36	3.8	6	Corpora lutea in regression and small follicles	0.41
12	BSH	12	3.0	4	Corpora lutea in regression and small follicles	0.37
13	ESH	24	2.0	4	Corpora lutea in regression and small follicles	0.26
14	BSH	24	4.1	2	Corpora lutea and small follicles	0.03
15	BSH	23	4.3	6	Corpora lutea and small follicles	0.02

AMH = anti-Müllerian hormone; BSH = British Shorthair; ESH = European Shorthair

AMH concentrations were also determined in 15 sexually intact cats presented for elective spaying or gynaecological examination and in 10 previously spayed cats presented because of orthopaedic issues in nine cases and in one case because of heat signs and cystic endometrial hyperplasia of the uterus after exogenous oestrogen administration. The group of sexually intact cats consisted of 13 European Shorthair, one Norwegian Forest Cat and one Birman cat. These cats were aged 6–48 months and had a body weight in the range of 2.3–6.1 kg. The group of previously spayed cats included seven European Shorthair, two Maine Coon and one Norwegian Forest Cat. These cats were aged 24–164 months and their body weight was in the range of 2.5–5.0 kg. Measurement of the AMH concentration was performed in all cats in serum samples collected for preanaesthesia blood testing or determination of the hormonal status.

### Ethical approval and informed consent

AMH determination in the cats that were not presented because of a suspected ORS or breeding soundness examination was conducted under the stipulations of the German Protection of Animals Act (reference number 55.2-1-54-2532-111-2016 from the Bavarian Government). All other examinations were carried out during routine diagnostics. All owners provided signed consent for the collection of data for the purpose of treatment and care of animals, as well as for research.

### Hormonal analysis

AMH measurements were performed at a commercial laboratory (Laboklin). AMH serum concentrations were determined using a chemiluminescence immunoassay on Cobas E602 analyser (Roche) using murine anti-AMH antibodies. The AMH test was validated for cats (intra-assay 1.8 %; inter-assay 7.4 %). Recovery of human AMH standard added to feline plasma showed changes in optical density parallel to the AMH standard curve. The minimum detection limit of the AMH test was 0.01 ng/ml and the maximum detection limit was 23 ng/ml.

Progesterone was measured with an automated enzyme linked fluorescent assay (MiniVidas; Biomerieux). Concentrations below 2 ng/ml were interpreted as baseline; concentrations above 2 ng/ml confirmed active luteal tissue.

### Histopathological examination

The removed tissue was measured and inspected grossly in detail with a focus on size, cut surface and colour. It was cut in slices and representative sites were embedded in paraffin according to standard procedures, sectioned at 3–4 μm and stained with haematoxylin and eosin (H&E).

### Statistical analysis

The statistical analysis was carried out with IBM SPSS 26.0 software. The data were checked for normal distribution using the Kolmogorov–Smirnov test. Since a normal distribution was not given, the non-parametric Kruskal–Wallis test with post hoc adjusting according to Bonferroni was used for group comparison. The data were visualised by using a dot plot with an overlying box plot. As specifications of the distribution the mean value, standard deviation, median and range of the metric parameters were determined. The level of significance was *P* <0.05.

## Results

Ultrasound examination confirmed the suspicion of remnant ovarian tissue in 11/13 cats (cats 1–3, 5–12, 14 and 15) (Table 1). In all of the vaginal swabs (cats 2, 3, 8, 9, 11, 14 and 15) the presence of superficial cells demonstrated a clinically relevant secretion of oestrogen. In one queen, the progesterone concentration was above 2 ng/ml at the time of first presentation (cat 14). The induction of ovulation in two cats resulted in a progesterone concentration above 2 ng/ml 6 days after treatment (cats 3 and 8).

The histopathological examination of the excised tissue (n = 14) confirmed that there was, in fact, remnant ovarian tissue. In 10 cases the ovarian tissue was located on the left side, in one queen on the right and in another one on both sides. In the two cases where cats underwent laparotomy outside the clinic, the exact location of ovarian tissue was not registered.

In 8/14 cats, the remnant ovarian tissue contained corpora lutea and follicles in different stages (cats 8–15). In cat 2 (Table 1) a whole ovary with corpora lutea in regression and small follicles was obtained. The ovarian tissue of the remaining five cats had diverse cystic alterations (cats 1 and 3–6).

**Figure 1:**
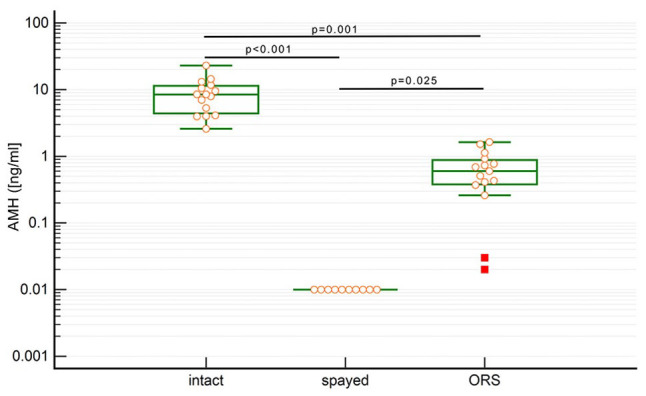
Box plot with an overlying dot plot of the anti-Müllerian hormone (AMH) values in sexually intact cats, spayed cats and cats with remnant ovarian tissue with a logarithmic y-axis indicating the AMH concentration in ng/ml. ORS = ovarian remnant syndrome

The results of the AMH determination in ORS cats (n = 15), ovariectomised cats (n = 10) and intact cats (n = 15) are shown in Table 2. All of the completely ovariectomised cats had an AMH concentration below the lower limit of the test (⩽0.01 ng/ml) (Figure 1). The mean AMH concentrations of the cats with ORS was significantly higher compared with the spayed cats (*P* = 0.025) and significantly lower than sexually intact cats (*P* = 0.001). Consequently, the mean AMH concentration of the sexually intact cats was significantly higher than that of the spayed cats (*P* <0.001). There was no overlapping of the AMH concentrations of the ORS cats with the sexually intact cats and with the ovariectomised cats.

**Table 2:** Minimum, maximum, median and mean anti-Müllerian hormone (AMH) concentrations with standard deviation in sexually intact cats (group 1), spayed cats (group 2) and cats with ovarian remnant syndrome (group 3)

Group	Number	AMH concentration in ng/ml			
		Minimum	Maximum	Median	Mean ± SD
1	15	2.59	⩾22.96	8.46	8.95 ± 5.25
2	10	⩽0.01	⩽0.01	⩽0.01	0.01 ± 0.00
3	15	0.02	1.63	0.60	0.67 ± 0.48

## Discussion

ORS is well known in cats as a result of incomplete removal of the ovaries during elective spaying, but the clinical diagnosis can be challenging.^1,2,4^

The suspicion for ORS arises when a previously spayed cat is presented with heat signs and the vaginal swab and/or serum oestrogen measurement confirms a clinically relevant oestrogen secretion. While most animals with ORS show signs of heat shortly after the initial spaying, the interval between spaying and heat signs can be as long as 10 years.^5^ In our study, 9/15 queens were presented within the first 6 months after spaying, and in one cat, the first heat signs occurred more than 5 years after spaying. It must be emphasised that heat signs in spayed cats can also be induced by exogenous oestrogens. ORS should not be based on oestrogen-induced alterations alone. One of the cats in the spayed group of this study was presented because of heat signs after spaying. Ultrasonography revealed signs of a cystic endometrial hyperplasia, but remnant ovarian tissue was not detected. The histopathological examination confirmed the ultrasonographic findings in the cat. In this case, the owner had used an oestrogen spray to reduce the side effects of her menopause. Exogenous oestrogens have the potential to induce behavioural and clinical signs of oestrogen up to and including alopecia in dogs.^15,22^ This case indicates that this is also possible in cats, and owners should use oestrogen-containing sprays or creams carefully and only on body parts that are not exposed to the animals. Further, oestrous-like behaviour has been associated with a hormonal active adrenocortical carcinoma in a spayed cat.^23^ Thus, the value of diagnostic approaches such as heat signs, vaginal smears or serum oestrogen determination depends on the definite exclusion of exogenous oestrogen application and endogenous extraovarian oestrogen sources.

An additional approach to diagnose ORS in queens with heat signs is the measurement of progesterone several days after induction of ovulation with hCG^11^ or gonadotropin-releasing hormone (GnRH). GnRH has not been ascertained in cats with ORS; however, the clinical use has shown its usability.^1,2^ When the queen is not under the influence of oestrogen at the time of presentation, oestradiol measurement after GnRH stimulation or a semi-quantitative quick test for LH have been described to verify the presence of ovaries, but these studies were conducted with intact and ovariectomised cats only.^13,14^

The ultrasonographic visualisation of the remnant ovarian tissue was successful in 13/15 cats in this study. Ultrasonography seems to be a valuable clinical method for the diagnosis of ORS, especially when combined with behavioural signs of heat or vaginal smears containing high amounts of superficial cells as described before.^15^ However, ORS should not be excluded when remnant ovarian tissue cannot be visualised during an ultrasound examination because the reliability of an ORS diagnosis with ultrasound depends on several factors: the equipment and the experience of the veterinarian; the hormonal activity of the ovarian tissue (eg, the formation of follicles, corpora lutea, cysts or tumours); and the size of the remnant ovarian tissue.^5^

It has been shown in dogs that AMH can be a useful tool to differentiate sexually intact bitches from spayed animals.^20,21^ This study shows that AMH is also useful in identifying ORS in queens. It is believed that only four cases of AMH determination in cats with ORS have been described so far.^16,18^ In three of these cases, the AMH serum levels in ORS cats have been in-between the levels of spayed and intact individuals, and in one case the AMH concentration has not been mentioned. In the present study, the AMH concentration differed significantly between completely spayed cats and cats with ORS as well as sexually intact individuals and ORS cats.

The highest AMH concentrations were measured in the cats with cystic ovarian alterations (cats 1 and 3–6) and in cat 2, which had a whole ovary left. In women, AMH has been described as a marker for polycystic ovarian syndrome with the highest sensitivity for anovulatory polycystic ovaries.^24^ In contrast, in dogs^25^ and cows,^26^ cystic ovarian alteration seems to have no impact on the AMH concentration. In cat 3, a luteal cyst combined with a high AMH concentration was found; however, it is unlikely that this type of cyst is the source of the elevated AMH concentration, because the origin of AMH is granulosa cells in follicles. Further research is needed to examine a possible correlation between ovarian cystic alteration and serum AMH concentration in the cat.

The two cats with the lowest AMH concentrations (cats 14 and 15) had active luteal tissue. In bitches with ORS it has been described that AMH may be low, when the remaining ovarian tissue contains mostly corpora lutea.^21^ This suggests that the AMH concentration may be low in cats, when the remnant ovarian tissue mainly consists of luteal tissue as described in dogs. Therefore, an additional progesterone measurement may be helpful for the diagnostic approach of the ORS, but further research is needed.

**Figure 2:**
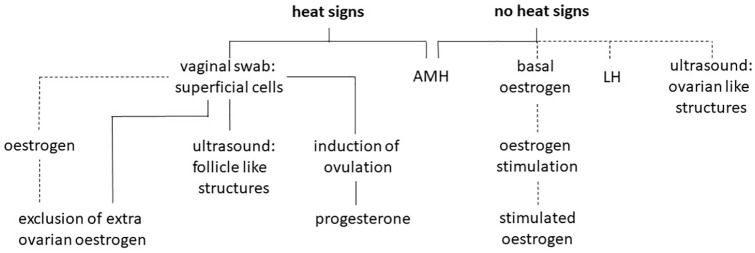
Diagnostic scheme for ovarian remnant syndrome in the cat. Prefered approach is shown in solid lines and others in dashed lines. AMH = anti-Müllerian hormone; LH = luteinising hormone

Every diagnostic approach should be as low stress as possible and the number of examinations, including blood sampling and drug administration, should be kept to a minimum. A possible scheme to diagnose ORS in cats including AMH measurement is shown in Figure 2. A first presentation during heat appears to be recommended. At this time oestrogen-induced changes can be seen in the gynaecological examination and follicular-like structures may be easier identified during ultrasonography. In addition, induction of ovulation can be performed during heat, and determination of progesterone several days later leads to a definite diagnosis. In addition, this study shows that a single blood sample and AMH determination helps to identify ORS in cats during heat as well as at any other time.

The treatment of choice for ORS is the surgical removal of the remnant ovarian tissue. Instead of conventional laparotomy with a considerably longer surgical incision than required for elective ovariectomy, laparoscopic treatment of ORS has also been described.^15^ However, abdominal adhesions and enlargement of the ovarian tissue due to pathological enlargement may complicate a laparoscopic procedure. In addition, hysterectomy cannot be performed via laparoscopy without enlargement of the incision.

In contrast to dogs, in which remnant ovarian tissue is most often found on the right side,^5^ there appears to be no preferred location in cats, which supports the findings of another report.^4^ In this study, the remnant ovarian tissue was found on the left side in 10 cases, on the right side in one case and on both sides in one case. It has been described that remnant ovarian tissue can revasculise in other abdominal locations such as the omentum or the peritoneum.^9^

In rare cases, ORS can be a result of anatomical specifics. In cat 2, a uterine horn aplasia combined with a renal agenesis was found on the left side. This is a previously described congenital abnormality with a likely predisposition in the Ragdoll,^27^ but also described in a domestic shorthair.^28^ A genetic influence seems possible because the occurrence has been described in littermates.^29^ Uterus unicornus may occur with or without renal agenesis, but all of the reported animals had two ovaries,^30^ and affected animals can become pregnant.^29^ The left ovary of the cat in this study was without an ovarian bursa or an oviduct and had a subjectively longer, thinner and flatter appearance than normal ovaries. Furthermore, the ovary seemed to be in a more cranial position and was more strongly attached to the dorsal peritoneum than the ovaries of a normal developed genital tract.

Female dogs with ORS seem to be predisposed to ovarian alterations, primarily granulosa cell tumours.^31,32^ This appears to be a rare finding in cats. Case reports described a luteoma^9^ and a granulosa cell tumour.^2^ A thecoma combined with behavioural signs of hyperandrogenism has also been described.^8,10^ None of the cats in this study had ovarian pathologies other than cystic alterations, and all of them showed typical oestrogen-induced heat signs at the time of presentation or reported from the owner.

## Conclusions

A single serum AMH determination is a useful diagnostic tool to identify cats with an ORS independent of the hormonal activity of the remnant ovarian tissue. Furthermore, serum AMH concentration enables the differentiation between intact queens and cats with ORS, which can be helpful in individuals with an unknown history.
